# Extended spectrum β lactamase-producing *Enterobacteriaceae* shedding by race horses in Ontario, Canada

**DOI:** 10.1186/s12917-020-02701-z

**Published:** 2020-12-09

**Authors:** Anat Shnaiderman-Torban, Shiri Navon-Venezia, Yossi Paitan, Holly Archer, Wiessam Abu Ahmad, Darryl Bonder, Erez Hanael, Israel Nissan, Gal Zizelski Valenci, Scott J. Weese, Amir Steinman

**Affiliations:** 1 The Robert H. Smith Faculty of Agriculture, Food and Environment, Koret School of Veterinary Medicine, The Hebrew University of Jerusalem, PO Box 12, 7610001 Rehovot, Israel; 2 Department of Molecular Biology, Faculty of Natural Science, Ariel University, Ariel, Israel; 3 Sackler Faculty of Medicine, Department of Clinical Microbiology and Immunology, Tel Aviv University, Tel Aviv, Israel; 4 Clinical Microbiology Lab, Meir Medical Center, Kfar Saba, Israel; 5 Department of Pathobiology, Ontario Veterinary College, University of Guelph, Guelph, Canada; 6 Hadassah Braun School of Public Health and Community Medicine, The Hebrew University of Jerusalem, Jerusalem, Israel; 7 Ontario Equine Hospital, Mississauga, Ontario Canada; 8 Ministry of Health, National Public Health Laboratory, Tel Aviv, Israel

**Keywords:** ESBL, Thoroughbred race horse, CTX-M

## Abstract

**Background:**

We aimed to investigate the prevalence, molecular epidemiology and prevalence factors for Extended Spectrum β-Lactamase-producing *Enterobacteriaceae* (ESBL-E) shedding by race horses. A cross-sectional study was performed involving fecal samples collected from 169 Thoroughbred horses that were housed at a large racing facility in Ontario, Canada. Samples were enriched, plated on selective plates, sub-cultured to obtain pure cultures and ESBL production was confirmed. Bacterial species were identified and antibiotic susceptibility profiles were assessed. *E. coli* sequence types (ST) and ESBL genes were determined using multilocus sequence type (MLST) and sequencing. Whole genome sequencing was performed to isolates harboring CTX-M-1 gene. Medical records were reviewed and associations were investigated.

**Results:**

Adult horses (*n* = 169), originating from 16 different barns, were sampled. ESBL-E shedding rate was 12% (*n* = 21/169, 95% CI 8–18%); 22 ESBL-E isolates were molecularly studied (one horse had two isolates). The main species was *E. coli* (91%) and the major ESBL gene was CTX-M-1 (54.5%). Ten different *E. coli* STs were identified. Sixty-four percent of total isolates were defined as multi-drug resistant. ESBL-E shedding horses originated from 8/16 different barns; whereas 48% (10/21) of them originated from one specific barn. Overall, antibiotic treatment in the previous month was found as a prevalence factor for ESBL-E shedding (*p* = 0.016, prevalence OR = 27.72, 95% CI 1.845–416.555).

**Conclusions:**

Our findings demonstrate the potential diverse reservoir of ESBL-E in Thoroughbred race horses. Multi-drug resistant bacteria should be further investigated to improve antibiotic treatment regimens and equine welfare.

**Supplementary Information:**

The online version contains supplementary material available at 10.1186/s12917-020-02701-z.

## Background

Antibiotic resistance has been described as an emerging concern in a wide range of pathogens, such as extended spectrum β-lactamase-producing *Enterobacteriaceae* (ESBL-E) [1, 2]. ESBL are widespread enzymes, which confer resistance to extended spectrum cephalosporins and aztreonam, not to cephamycins or carbapenems, and are inhibited by β-lactamase inhibitors [3]. These genes are mostly mobile genetic element encoded, and may carry additional antimicrobial resistances, including aminoglycoside, sulfa-derivative, trimethoprim and quinolone resistance [4]. Therefore, treatment options are limited. In humans, infections with ESBL-E are associated with increased morbidity, mortality, length of hospital stay, delay of targeted appropriate treatment and higher costs [5].

Penicillins and cephalosporins are commonly prescribed in horses [6] and ESBL-E were reported as colonizing and infecting horses. In a longitudinal study in an equine clinic in the UK, increasing carriage rates were reported over a decade [7]. Reported infections in horses include wounds, respiratory, urinary tract and umbilical infections [8–10]. Race horses represent a unique equine community, due to high population density, shared training and living facilities, intense movement of horses, stress and medical treatments. All these factors create complex epidemiology for pathogen transfer [11]. Infectious diseases and bacterial pathogens specifically, have a major impact in the race horses industry leading to high rates of antibiotic use. Frequent inappropriate use of antimicrobials in race horses has been documented previously and implied that a greater scrutiny should be applied when determining whether antimicrobials are indicated in the treatment of horses with poor athletic performance [12]. Studies investigating antibiotic resistant bacteria in race horses are limited and have most often involved *Enterococcus sp*. [13], methicillin-resistant staphylococci [14] and *E. coli* [15]. A recent study detected 8.2% shedding rate of ESBL-producing *E. coli* in race horses [16]. However, data regarding colonization of race horses with different ESBL-E species, sequence types (ST) and factors associated with carriage are lacking.

In this study, we aimed to determine the ESBL-E shedding in Thoroughbred race horses, to identify the bacterial species, STs and ESBL genes, and to define the prevalence factors associated with ESBL-E shedding. We hypothesized that Thoroughbred race horses shed ESBL-E and that antibiotic treatment is a prevalence factor for shedding.

## Results

### Characterization of equine population

**Fig. 1:**
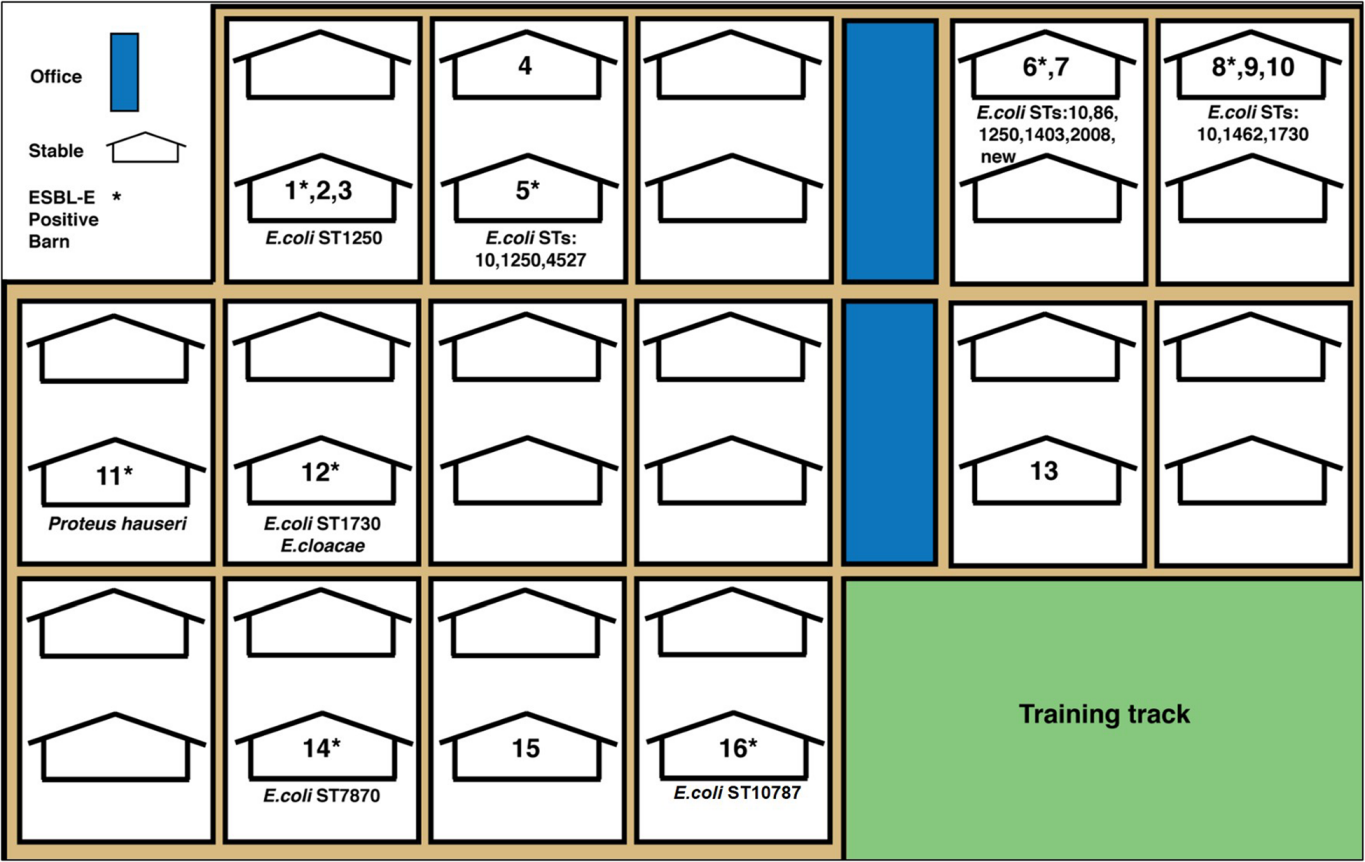
Stables area map. Sampled barns are numbered (each stable contains more than one barn). A barn number marked with asterisk represent an ESBL-E positive barn. ESBL-E species are described under each specific barn

The study population included Thoroughbred horses originating from 16 different barns (Fig. 1), each barn trained by a different trainer. Between 2 and 35 horses were sampled per barn (mea*n* = 11, median = 9), based on the owner’s or trainer’s permission, number of horses in the barn and number of horses from which feces were available. The classification of eight sampled horses to their specific barn was unavailable. Data regarding the identity of the barn’s veterinarian were available for 13 barns and included five different veterinarians. Medical records, other than signalment, were available for 56% of horses (*n* = 94/169). Age data were available for 104 horses, and the age ranged between two to 11 years, with a median of 3 years. Sex was recorded in 103 horses, including 46% females (*n* = 47/103), 42% geldings (*n* = 43/103) and 12% stallions (*n* = 13/103). Recent pathologies included orthopedic diseases, respiratory diseases, dermatological lesions, metabolic diseases, open wounds, colic, ophthalmic diseases and teeth abnormalities (Table 1).

**Table 1:** Univariable analysis for extended-spectrum β-lactamase-producing *Enterobacteriaceae* shedding by Thoroughbred race horses

Variable	classification	Frequency ,% of horses	***P*** value	Prevalence OR (95% CI)
Sex			0.226	
	Female	46 (*n* = 47/103)	Reference	
	Geldings	42 (*n* = 43/103)	0.739	2.365 (0.316–5.063)
	Stallion	12 (*n* = 13/103)	0.095	4.005 (0.784–20.466)
Veterinarian			0.741	
	1	39.8(*n* = 45/113)	Reference	
	2	3.5 (*n* = 4/113)	a	
	3	8.8 (*n* = 10/113)	0.488	0.426 (0.038–4.752)
	4	45 (*n* = 52/113)	0.623	0.692 (0.159–3.001)
	5	1.8 (*n* = 2/113)	a	
Age	3 (2–11 years)		0.513	1.114 (0.805–1.545)
Pathologies one month prior to sampling			0.369	
	Orthopedic diseases	27 (*n* = 24/89)	0.273	0.381 (0.067–2.137)
	Respiratory diseases	20 (*n* = 18/89)	0.511	0.554 (0.095–3.223)
	Dermatological lesions	6 (*n* = 5/89)	0.828	0.703 (0.029–16.716)
	Metabolic diseases	3 (*n* = 3/89)	0.133	112.278 (0.467–322.807)
	Open wounds	1 (*n* = 1/89)	a	
	Colic	0		
	Ophthalmic diseases	0		
	Teeth abnormalities	0		
Pathologies two-three months prior to sampling			0.494	
	Orthopedic diseases	34 (*n* = 31/91)	0.689	0.764 (0.204–2.86)
	Respiratory diseases	29 (*n* = 26/91)	0.172	0.306 (0.0557–1.675)
	Dermatological lesions	3 (*n* = 3/91)	0.643	1.833 (0.141–23.825)
	Metabolic diseases	2 (*n* = 2/91)	a	
	Open wounds	0		
	Colic	1 (*n* = 1/91)	a	
	Ophthalmic diseases	0		
	Teeth abnormalities	0		
Pathologies three-six months prior to sampling			0.181	
	Orthopedic diseases	33 (*n* = 27/81)	0.138	0.3 (0.061–1.472)
	Respiratory diseases	37 (*n* = 30/81)	0.102	0.266 (0.054–1.3)
	Dermatological lesions	2 (*n* = 2/81)	a	
	Metabolic diseases	1 (*n* = 1/81)	a	
	Open wounds	0		
	Colic	0		
	Ophthalmic diseases	1 (*n* = 1/81)	a	
	Teeth abnormalities	1 (*n* = 1/81)	a	
Pathologies six-twelve months prior to sampling			0.544	
	Orthopedic diseases	27 (*n* = 15/56)	0.715	0.708 (0.111–4.51)
	Respiratory diseases	23 (*n* = 13/56)	0.379	0.354 (0.035–3.577)
	Dermatological lesions	0		
	Metabolic diseases	0		
	Open wounds	0		
	Colic	4 (*n* = 2/56)	0.341	4.249 (0.216–83.513)
	Ophthalmic diseases	4 (*n* = 2/56)	a	
	Teeth abnormalities	5 (*n* = 3/56)	a	
Treatments and procedures one month prior to sampling	Antibiotic treatment	11.1 (*n* = 10/90)	**0.006**	**11.411 (1.991–65.395)**
	Omeprazole treatment	13 (*n* = 12/91)	0.721	1.353 (0.257–7.115)
	Anti-inflammatory drugs	46.8 (*n* = 44/94)	0.352	0.519 (0.131–2.062)
	Anti-parasitic drugs	2.2 (*n* = 2/92)	0.228	5.764 (0.339–98.034)
	Food additives	7.5 (*n* = 7/93)	0.778	0.642 (0.029–14.125)
	Naso-gastric tube insertion	1.1 (*n* = 1/92)	a	
	Endoscopy	13.8 (*n* = 13/94)	0.479	1.675 (0.401–6.992)
	Hospitalization	6.5 (*n* = 6/92)	0.233	3 (0.494–18.222)
	Surgical procedure		0.128	
	Castration	5.5 (*n* = 5/91)	0.128	4.364 (0.654–29.127)
	Arthroscopy	1.1 (*n* = 1/91)	a	
Treatments and procedures Two-three months prior to sampling	Antibiotic treatment	14.6 (*n* = 13/89)	0.378	1.919 (0.449–8.203)
	Omeprazole treatment	17.77 (*n* = 16/90)	0.864	0.868 (0.172–4.388)
	Anti-inflammatory drugs	55.9 (*n* = 52/93)	0.101	0.253 (0.048–1.31)
	Anti-parasitic drugs	7.7 (*n* = 7/91)	0.342	2.336 (0.406–13.443)
	Food additives	12.1 (*n* = 11/91)	0.809	1.22 (0.235–6.378)
	Naso-gastric tube insertion	3.3 (*n* = 3/91)	a	
	Endoscopy	23.1 (*n* = 21/91)	0.487	0.569 (0.116–2.792)
	Hospitalization	4.4 (*n* = 4/91)	a	
	Surgical procedure		a	
	Castration	2.25 (*n* = 2/89)	a	
	Arthroscopy	2.25 (*n* = 2/89)	a	
Treatments and procedures three- six months prior to sampling	Antibiotic treatment	18.2 (*n* = 14/77)	0.392	0.392 (0.046–3.348)
	Omeprazole treatment	15.58 (*n* = 12/77)	0.722	1.358 (0.252–7.332)
	Anti-inflammatory drugs	64.6 (*n* = 51/79)	0.088	0.241 (0.047–1.237)
	Anti-parasitic drugs	10.1 (*n* = 8/79)	0.445	1.966 (0.347–11.146)
	Food additives	6.4 (*n* = 5/78)	0.79	1.364 (0.139–13.381)
	Naso-gastric tube insertion	1.3 (*n* = 1/78)	a	
	Endoscopy	26.3 (*n* = 21/80)	0.87	0.889 (0.216–3.657)
	Hospitalization	6.5 (*n* = 5/77)	0.79	1.634 (0.139–13.381)
	Surgical procedure		a	
	Castration	5.3 (*n* = 4/76)	a	
	Arthroscopy	1.3 (*n* = 1/76)	a	
Treatments and procedures Six-twelve month prior to sampling	Antibiotic treatment	12.1 (*n* = 7/58)	0.622	1.8 (0.174–18.638)
	Omeprazole treatment	10.5 (*n* = 6/57)	0.135	4.5 (0.626–32.39)
	Anti-inflammatory drugs	50 (*n* = 29/58)	0.482	0.576 (0.124–2.679)
	Anti-parasitic drugs	8.6 (*n* = 5/58)	0.522	2.19 (0.198–24.117)
	Food additives	1.7 (*n* = 1/58)	a	
	Naso-gastric tube insertion	12.1 (*n* = 7/58)	0.984	1.024 (0.107–9.838)
	Endoscopy	17.2 (*n* = 10/58)	0.784	0.732 (0.079–6.795)
	Hospitalization	0	a	
	Surgical procedure		a	
	Castration	1.7 (*n* = 1/59)	a	
	Arthroscopy	0		

^a^ No positive ESBL-E cases in the category, therefore *p*-value and prevalence OR cannot be calculated

Data regarding antibiotic treatment were obtained for 54% of horses (*n* = 91/169), of which 35% (*n* = 32/91) had been treated with an antibiotic at least once within the past year. Antibiotic treatments varied and included trimethoprim-sulpha, gentamicin, enrofloxacin, penicillin, ceftiofur, oxytetracycline and different combinations of these antibiotics. Data regarding omeprazole treatment was obtained for 54% of horses (*n* = 91/169), of which 35% (*n* = 32/91) were treated at least once within the past year. Data regarding anti-inflammatory treatment was obtained for 56% of horses (*n* = 95/169), of which 77% (*n* = 73/95) were treated at least once within the past year. Data regarding hospitalization and surgical procedures was obtained for 54% of horses (*n* = 92/169), of which 15% (*n* = 14/92) were hospitalized and/ or underwent a surgical procedure at least once within the past year (all parameters are divided to four time categories, as indicated in Table 1).

### Prevalence of ESBL-E shedding

Twelve percent (*n* = 21/169) of the horses from 8/16 (50%) barns shed at least one ESBL-E strain. One horse shed two strains (Table 2). Overall, 22 ESBL-E were recovered. The majority of the ESBL-E isolates (*n* = 20/22, 91%) were *E. coli,* while the other ESBL-E isolates were identified as single isolates each of *Enterobacter cloacae* and *Proteus hauseri*.

**Table 2:**
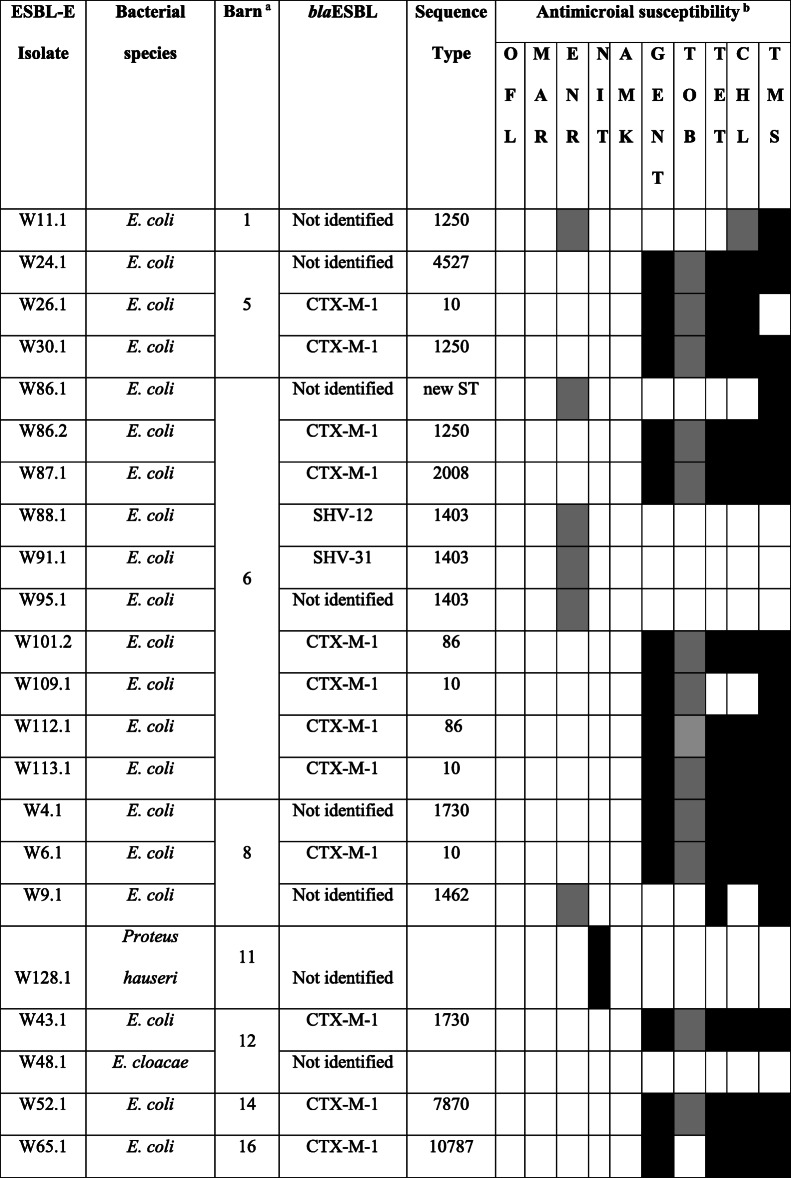
Characteristics and antibiograms of 22 ESBL-E isolates

^a^ There was no statistical difference in prevalence rate between barns. Barn no. 6 had the largest number of isolates due to the largest number of sampled horses

^b^ All isolates were resistant to ampicillin, piperacillin, cephalosporins and susceptible to imipenem. A black box indicates resistance, grey box indicates intermediate resistance and a white box indicates susceptibility

*AMK* amikacin, *CHL* chloramphenicol, *ENR* enrofloxacin, *GENT* gentamicin, *MAR* marbofloxacin, *NIT* nitrofurantoin, *OFL* ofloxacin, *TET* tetracycline, *TMS* trimethoprim sulpha, *TOB* tobramycin

### Antibiotic susceptibility profiles, STs and *bla*ESBL-genes

Antibiotic resistance rates of ESBL-E isolates were: 73% for trimethoprim-sulpha, 64% for tetracycline and gentamicin, 60% for chloramphenicol and 5% for nitrofurantoin. All isolates were resistant to ampicillin, piperacillin and cephalosporins (cefalexin, cefpodoxime, cefovecin and ceftifour), as well as susceptible to amikacin, ofloxacin, marbofloxacin and imipenem (Table 2). Sixty-eight percent of isolates (*n* = 15/22) were identified as multi-drug resistant (MDR).

Ninty-five percent of *E. coli* (*n* = 19/20) belonged to ten different STs and one *E. coli* isolate possessed a new combination of alleles suggesting a new *E. coli* ST. Ten STs included (Table 2): ST10 (a horse from barn 5, two horses from barn 6 and a horse from barn 8), ST1403 (three horses from barn 6), ST1730 (one horse from barn 8 and one horse from barn 12), ST1250 (one horse from barn 1, one horse from barn 5 and one horse from barn 6), ST86 (two horses from barn 6), ST1462 (one horse from barn 8), ST4527 (one horse from barn 5), ST7870 (one horse from barn 14), ST2008 (one horse from barn 6) and ST10787 (one horse from barn 16). Neither of the isolates belonged to the worldwide ESBL-producing *E. coli* ST131 lineage. The major ESBL gene found was *bla*_CTX-M-1_ (*n* = 12/22, 54.5%, Table 2).

### Factors associated with ESBL-E shedding

In a mixed effects logistic regression, in which the barn was considered the random effect, shedding of ESBL-E by an individual animal was found to be significantly associated with antibiotic treatment within a month prior to sampling (*p* = 0.006, Table 1). Due to the small sample size, we chose to include the three most clinically relevant variables in the multivariable model [17]. Therefore, the model included the following parameters: antibiotic treatment within a month prior to sampling, surgery within a month prior to sampling (including two categories: castration and arthroscopy) and pathologies during three to six months prior to sampling (including two categories: respiratory and orthopedic lesions) (*p* ≤ 0.2, Table 3). The final model included 71 horses. The only significant prevalence factor in the model was antibiotic treatment within a month prior to sampling (*p* = 0.016, prevalence OR = 27.72, 95% CI 1.845–416.555, Table 3).

**Table 3:** Multivariable analysis for extended-spectrum β-lactamase-producing *Enterobacteriaceae* shedding by Thoroughbred race horses

Variable	classification	***P*** value	Prevalence OR (95% CI)
Antibiotic treatment in the previous month		**0.016**	27.72 (1.845–416.555)
Surgical procedure in the previous month	Castration	0.4	0.353 (0.031–3.992)
	Arthroscopy	a	
Pathologies in the previous month	Respiratory	0.526	0.506 (0.061–4.161)
	Orthopedic lesions	0.751	0.727 (0.101–5.237)

^a^ No positive ESBL-E cases in the category, therefore p-value and prevalence OR cannot be calculated

## Discussion

Extended-spectrum β-lactamase-producing *Enterobacteriaceae* shedding by Thoroughbred race horses was investigated in this study, bacterial strains were characterized and a prevalence factor was determined.

Twenty-one out of 169 sampled horses (12%, 95% CI 8–18), originating from 16 different barns, shed ESBL-E. The prevalence of shedding horses varied between barns (zero to 33%), although there was no significant difference between barns, due to differences in the number of sampled horses. The difference in prevalence rates may be explained by the diversity of sampled facilities, representing different working environments (trainers, cleaners and veterinarians), different antibiotic use practices and transmission between barns. The overall prevalence we found in this study is higher compared to the rate of shedding ESBL-producing *E. coli* in an earlier study of UK equine farms [1] and similar to shedding rate in race horses with MDR *E. coli* reported from Korea [13]*.* In order to better understand whether ESBL-E colonization rate is significantly higher in race horses compared to farm horses, a cross sectional study should compare these two equine cohorts within the same country.

Drug resistance genes in general, and especially ESBL genes may originate from the environment, including manure, air and other animals. In recent studies, CTX-M-15 was identified as an emerging enzyme in human and in veterinary medicine in general and specifically in horses [7], but was not identified in this study. Drug resistance genetic variation may be also influenced by different facilities and interfaces [18]. The fact that a common gene, CTX-M-1, was detected in most horses (57%, *n* = 12/21) may also indicate on a common environmental source.

A high ESBL-E prevalence rate in Thoroughbred race horses may be a consequence of high stress and intensive interface, including a high number of horses housed and trained in the same facility and a frequent medication usage, especially antibiotics. A recent study on healthy farm horses revealed that more than five people taking care of horses daily, as well as the presence of an horse that has been treated medically on the last three months in the farm, were associated with ESBL/ Ampicillin C beta lactamase (AmpC) carriage [19]. These factors are relevant to the intensive care of race horses as well. A high prevalence rate in race horses has broader implications considering human health, animal health and welfare, as well as an ecological impact. Resistant bacterial dissemination may influence farm personnel, as owning or a contact with a horse was associated with ESBL-E carriage in humans [20]. Infections with these MDR bacteria limit treatment options; thereby affect both human and horse health and welfare. In this study, ESBL-E were investigated and detected in manure, but in a recent study it has also been detected in air samples and nostril swabs in farm horses [18]. A possible environmental ESBL-E contamination, originated from intensive equine facilities, may have an important ecological impact. Therefore, the importance of ESBL-E prevalence in these facilities is crucial.

The major ESBL-E species was *E. coli* (91%), followed by *Enterobacter cloacae* and *Proteus hauseri* (one isolate each). *E. coli* isolates belonged to multiple sequence types, which varied between and within the same facility. According to a previous study, intra- and inter-horse genetic diversity exists among *E. coli* strains and horizontal transfer and/ or selection of resistance genes probably occur within the equine gut microbiome [21]. Therefore, in our study, we suggest that the existence of diverse sequence types occurs due to horizontal transfer of β-lactamase genes and plasmids rather than clonal spread.

Among the ESBL-producing *E. coli* isolates recovered, we identified two *E. coli* strains, which are known to also infect people; ST10 and ST86. *E. coli* ST10 was identified before as a very common ST, isolated from human urinary tract infection cases and food-animal reservoirs in Canada [22], Europe [23] and the United States [24]. *E. coli* ST86 was also reported as a human pathogen, causing diarrhea and bacteremia [25, 26]. Both ST10 and ST68 were also isolated from horses in equine clinics in Europe, as well as ST1250 and ST2008 [9, 27, 28]. ST1730 has been also isolated before from equine clinical samples [28]. Overall, we identified some colonizing strains and some resistant zoonotic pathogens, which may pose a potential transmission risk to equine handlers. Five *E. coli* STs (ST10, ST1403, ST250, ST1730 and ST86) occurred in different barns during the study period suggesting inter-barn transmission. In barns 5, 6 and 8, multiple horses shed ESBL-producing *E. coli*, each colonized with a different ST (three to five different STs).

Moreover, the most prevalent ESBL gene we identified was CTX-M-1. In the last decade, the CTX-M gene family has become dominant and involved in many antibiotic resistant human infections in complicated community patients, usually with underlying disease, recent antibiotic usage or healthcare contact [29]. In equine studies, *bla*CTX-M-1 group was also reported as the most prevalent gene group associated with ESBL-production [9]. Transmission of plasmid carrying *bla*CTX-M-1 between commensal *E. coli* in pigs and farm workers was demonstrated before [30], a finding which reinforces the potential zoonotic risk. A further study should investigate the link between human-equine contact and bacterial transfer, as well as the duration of carriage following antibiotic treatment, in order to establish appropriate guidelines.

In light of our findings and the lack of data regarding ESBL-E in race horses, there is a great importance in identifying factors associated with ESBL-E shedding. We determined that individual antibiotic therapy is associated with ESBL-E shedding (*p* = 0.016), as described before both in dogs [31] and humans [32]. We did not identify any specific antibiotic agent which was significantly associated with shedding, probably due to multiplicity of agents in use. Therefore, a horse treated with antibiotics may constitute a risk for resistant bacterial transfer to its environment for an undetermined period of time. In addition, we revealed a variety of antimicrobials used in Thoroughbred race horses, including third generation cephalosporins and quinolones. A previous study revealed inappropriate use of antibiotics in race horses [12], which may explain the variety of antibiotics in use. We also identified that most of the isolates were MDR (68%, *n* = 15/22). In this study, we did not investigate clinical samples, or the connection between shedding and infection, which was demonstrated in human medicine before [33]. A further study should focus on clinical samples and inquire the connection between antibiotic treatment, ESBL-E shedding and infection in race horses, which has a financial significance.

Limitations of this study include convenience sampling and a relatively small sample size, which affect the ability to generalize these results. In this study, we were limited in sample size due to owners and trainers permission. In future studies, larger number of horses in more barns, should be selected randomly, which could result in higher statistical power, not only based on their availability on the sampling dates, but this must be determined in advance. Furthermore, it would be better to sample horses in more than one race-track and preferably in more than one country.

Another limitation is that this was a cross-sectional study and therefore descriptive and not longitudinal or cohort. Cross-sectional study are limited and cannot determine which variable is the cause and which the effect. For example, the finding that shedding of ESBL-E by an individual animal was found to be significantly associated with antibiotic treatment within a month prior to sampling. However, there is no guarantee that the ESBL-E bacteria were not present prior to antibiotic use, or even that clinical signs associated with ESBL-E did not lead to antibiotic use. Had this been a cohort study, the horses would have been verified to have been ESBL-E-free at the beginning of the study, which could have then led to the determination of risk factors influencing bacterial infection.

The use of retrospective medical data collection was another limitation of this study. This resulted in limited availability of medical records, which were only available for little over half of the horses. This further limit our ability to draw generalized conclusions and reveal associations since it lowered the sample size of the available information. A prospective study design in the future will enable to collect data for all horses and will enable to draw better conclusions.

## Conclusion

The results from this study substantiate the occurrence of ESBL-E in Thoroughbred race horses. Our data confirms that Thoroughbred race horses may shed MDR ESBL-E, some of which may pose a zoonotic hazard. Recommendations for decreasing antimicrobial resistance among horses consist on implementation of antibiotic stewardship principle, including veterinary involvement, culture and sensitivity results, proper doses and overall decreased reliance on antimicrobials [34]. Further studies and active surveillance should focus on understanding ESBL-E epidemiology and transmission routes.

## Methods

### Equine study population, study design and sampling methods

This study was performed in a large racing facility in Ontario, Canada. The facility houses over 2000 horses from a large number of different trainers in 39 barns. Many horses move off-site regularly to race at other facilities or for other purposes.

Sample size was calculated (WinPepi, version 11.62) based on the assumption that the estimated prevalence of ESBL-production among potential pathogens in race horses is 10% [15], with power of 80%, and acceptable difference of 5%, resulting in minimal sample size of 139 horses. A convenience sample of horses was evaluated. All horses at the facility on the day of sampling were eligible for inclusion. Fresh fecal samples were collected during two sampling days in October 2017. Samples were collected from freshly passed feces in stalls, and only if it could be attributed to a specific horse. The number and selection of sampled animals at each barn was determined by owner’s or trainer’s permission. Overall, 169 fecal samples from 169 horses were collected. The study was performed in compliance with institutional guidelines for research on animals and an informed owner consent was obtained. Since we did not actually use horses an ethics approval was not required.

### Equine demographic and medical data

Equine demographics and medical records were reviewed for the following information: signalment (age, sex and breed), veterinarian, barn/ trainer, pathologies, antibiotic treatment, omeprazole treatment, anti-inflammatory and anti-parasitic treatment, food additives, insertion of a nasogastric tube, endoscopy, hospitalization and surgical procedures. These parameters were recorded in four categories, depending on the time of treatment prior to sampling, (i) ≤ one month (ii) two to three months (iii) three to six months and (iv) six to twelve months.

### Extended-spectrum β-lactamase-producing *Enterobacteriaceae* isolation and species identification

Fecal samples were collected in sterile containers and inoculated directly into an enrichment broth (BD Difco Luria Bertoni infusion enrichment broth, Becton Dickinson and Company, Sparks, USA) to increase sensitivity of ESBL-E detection [35]. After incubation at 37 °C (18-24 h), enriched samples were plated onto selective plates (Chromagar ESBL plates, Biomerieux Canada, St Laurent, Canada) and incubated at 37 °C for 24 h. When more than one colony was present, multiple colonies were isolated and tested only if they appeared different. Pure isolates after sub-culturing were stored at − 80 °C stocks for further analysis.

All isolates were subjected to analysis by Matrix-assisted laser desorption/ionization time-of-flight mass spectrometer (MALDI-TOF^,^ Microflex LT., Bruker Daltonics Ltd) for species identification and to vitek-2 analysis (BioMérieux, Inc., Marcy-l’Etoile, France) for antibiotic susceptibility testing using GN65 Vitek 2 card. Resistance rates reported were considered as complete resistance (excluding intermediate resistance). ESBL production was confirmed by clavulanic acid combination disk diffusion using cefotaxime and ceftazidime discs with and without clavulanic acid (BD BBL, Becton Dickinson and Company, Sparks, USA). Results were interpreted according to the Clinical and Laboratory Standards Institute (CLSI) guidelines [36]. MDR bacteria were defined as such due to their in vitro resistance to three or more classes of antimicrobial agents [37].

### Molecular characterization of ESBL-E

Isolates were examined for presence of the *bla*CTX-M ESBL groups using a multiplex polymerase chain reaction (PCR) from ESBL-E DNA lysates [37]. PCR was performed in a 10 μl mixture consisting of 2 μl GoTaq® Green Master Mix (Promega, Madison, USA), 0.3 μl DNase/RNase-free water (Promega, Madison, USA), 0.3 μl of each primer *bla*CTX-M-1, 1 μl of each primer *bla*CTX-M-2, 0.5 μl of each primer *bla*CTX-M-9 and *bla*CTX-M-25 (10 μM), 0.1 μl MgCl_2_ (50 mM) and 1 μl DNA template. PCR was carried under the following conditions: an initial 2 min denaturation at 94 °C, followed by 29 cycles of 30 s at 95 °C, 15 s at 61.7 °C and 30 s at 72 °C, with a final elongation at 72 °C for 5 min.

Isolates that were found to be *bla*CTX-M PCR negative were further examined for the presence of *bla*OXA-1, *bla*OXA-2, *bla*OXA-10 [38], *bla*TEM and *bla*SHV groups [39]. PCR for *bla*OXA genes was performed in different reactions (not multiplex) of 10 μl mixture consisting of 5 μl GoTaq® Green Master Mix, 3.6 μl DNase/RNase-free water, 1 μl of each primer and μl DNA template. PCR was carried under the following conditions: an initial 2 min denaturation at 94 °C, followed by 29 cycles of 30 s at 95 °C, 30 s at 51 °C/ 56 °C/ 55 °C for OXA-1/ OXA-2/ OXA-10 respectively and 60 s at 72 °C, with a final elongation at 72 °C for 5 min.

PCR for *bla*TEM and SHV genes was performed in two reactions of 50 μl mixture consisting of 25 μl GoTaq® Green Master Mix, 20 μl DNase/RNase-free water, 2 μl of each primer (Table S1) and 1 μl DNA template. PCR was carried under the following conditions: an initial 2 min denaturation at 95 °C, followed by 39 cycles of 95 °C for 1 min, 15 s at 52 °C and 50 s at 72 °C, with a final elongation at 72 °C for 5 min. PCR products were purified using a PCR purification kit, sequences were analyzed and compared with NCBI database to identify the ESBL gene allele.

All ESBL-producing *E. coli* Isolates were genotyped using an Enterobacterial repetitive intergenic consensus (ERIC) PCR amplification [40]. PCR was performed a 15 μl mixture consisting of 0.15 μl HIFI enzyme, 3 μl HIFI buffer (PCRBIOSYSTEMS, London, UK), 9.35 μl DNase/RNase-free water, 1.5 μl of ERIC2 primer and 1 μl DNA template. PCR was carried under the following conditions: an initial 30 s denaturation at 98 °C, followed by 34 cycles of 98 °C for 10 s, 30 s at 52 °C and 5 min seconds at 72 °C, with a final elongation at 72 °C for 10 min. Results were analyzed (GelJ, Java application) and strains showing a distinct ERIC PCR pattern were genotyped by multi locus sequence type (MLST) analysis as previously described using the Achtman scheme (IDGenomics, Seattle, USA) [41]. ST of two *E. coli* isolates (W65.1 and W112.1), for which ST were not identified by PCR- based MLST scheme, were determined *in-silico* using whole genome sequencing (WGS) data.

### Whole genome sequencing

Twelve isolates, which were recognized as blaCTX-M-1 group-producers, were subjected for WGS, in order to determine *in-silico* the ST and the *bla*CTX-M-1 allele. The isolates were inoculated into LB agar with ampicillin (100 μg/m) at 37 °C for overnight incubation. Typical colonies from the fresh overnight culture were re-suspended in saline to about 1.5 OD600 nm. Four hundred microliter from each strain suspension was used for DNA extraction using MagNA Pure Compact (Roche) according to the manufacturer instructions. The DNA purity was determined using NaNodrop 2000 spectrophotometer and the amount of dsDNA was measured using DeNovix dsDNA High Sensitivity Kit (Cat# KIT-DSDNA-HIGH-2) and DeNovix QFX Fluorometer. DNA libraries were prepared using a Nextera XT DNA Library Preparation Kit following the manufacturer’s instructions. Sequencing was performed on an Illumina MiSeq platform using a 250-bp paired-end read v2 kit. Bioinformatics analyses were performed using the PATRIC v3.5.36 platform, including verification of the MLST profiles and CTX-M-1 genes [42]. The identification of CTX-M-1 gene alleles was confirmed by ResFinder-3.2 (https://cge.cbs.dtu.dk/services/ResFinder/).

### Statistical analysis

Descriptive statistics were performed for all variables and are given as percentages, mean ± standard deviation or medians (range) (IBM Statistics SPSS, version 24.0).

To account for repeated measures within barns, logistic regression mixed-effect, with random effect at the barn-level, was used to explore the associations between the medical and demographic data and ESBL-E shedding.

Four variables (sex, veterinarian, pathologies and surgery) had more than two levels for comparison. The following variables were coded as yes/no (binary variables): antibiotic treatment, omeprazole usage, anti-inflammatory drugs usage, food additives, treatment with naso-gastric tube, endoscopy and hospitalization. These variables were evaluated at four different time frames (Table 1). Antibiotic treatment was coded as yes/no, due to variety of drugs and drug combination used (resulting in 10 different categories). The independent variables are stated in Table 1, whereas the dependent variable was ESBL-E shedding. A value of *P* ≤ 0.05 was considered significant. Multivariable analysis was conducted using selected variables with *P* ≤ 0.2 (StataCorp. 2017. *Stata Statistical Software: Release 15*. College Station, TX: StataCorp LLC).

### Availability of data

This Whole Genome Shotgun project has been deposited at DDBJ/ENA/GenBank under the Bio-Project accession number PRJNA612199.

## Supplementary Information

**Additional file 1: Table S1.** PCR primers and reactions conditions

## Data Availability

WGS data is available in the Genbank (Bio-Project accession number PRJNA612199), and the datasets used and analyzed during the current study are available from the corresponding authors on reasonable request.

## Abbreviations

AmpC: Ampicillin C beta lactamase
CHL: Chloramphenicol
CI: Confidence interval
CLSI: Clinical and Laboratory Standards Institute
CTX-M: Cefotaxime hydrolyzing capabilities
ENR: Enrofloxacin
ERIC: Enterobacterial repetitive intergenic consensus
ESBL: Extended spectrum β lactamase
ESBL-E: Extended spectrum β lactamase-producing *Enterobacteriaceae*
GENT: Gentamicin
MALDI-TOF: Matrix-assisted laser desorption/ionization time-of-flight mass spectrometer
MAR: Marbofloxacin
MDR: Multi-drug resistance
MLST: Multi locus sequence type
NIT: Nitrofurantoin
OFL: Ofloxacin
OXA: Oxacillin hydrolyzing capabilities
SHV: Sulfhydryl variable (enzyme’s active site)
SPSS: Statistical package for the social sciences
ST: Sequence type
TEM: Temoneira
TET: Tetracycline
TMS: Trimethoprim sulpha
TOB: Tobramycin
