# Supplementary material for: Extended spectrum β lactamase-producing Enterobacteriaceae shedding by race horses in Ontario, Canada
Source: BMC Vet Res. 2020 Dec 9;16:479. doi: 10.1186/s12917-020-02701-z (PMC7726890; doi:10.1186/s12917-020-02701-z)
Supplement: Supplementary file 1 — Additional file 1: Table S1. PCR primers and reactions conditions [file 12917_2020_2701_MOESM1_ESM.docx]

Table S1: PCR primers and reactions conditions

| Primer | Sequence (5'-3') | Target | Size of  product | Reference | |
| --- | --- | --- | --- | --- | --- |
| CTX-M-1grF | AAAAATCACTGCGCCAGTTC | CTX-M-1  group | 415 | [42] |  |
| CTX-M-1grR | AGCTTATTCATCGCCACGTT |  |  |  |  |
| CTX-M-2grF | CGACGCTACCCCTGCTATT | CTX-M-2  group | 552 |  |  |
| CTX-M-2grR | CCAGCGTCAGATTTTTCAGG |  |  |  |  |
| CTX-M-9grF | CAAAGAGAGTGCAACGGATG | CTX-M-9  group | 205 |  |  |
| CTX-M-9grR | ATTGGAAAGCGTTCATCACC |  |  |  |  |
| CTX-M-8grF | TCGCGTTAAGCGGATGATGC | CTX-M-8  group | 666 |  |  |
| CTX-M-25grF | GCACGATGACATTCGGG | CTX-M-25  group | 327 |  |  |
| CTX-M-8/25grR | AACCCACGATGTGGGTAGC | CTX-M-8/ 25  group |  |  |  |
| OXA-1-F | AGCCGTTAAAATTAAGCCC | *bla*OXA-1 | 908 | [38] |  |
| OXA-1-R | CTTGATTGAAGGGTTGGGCG |  |  |  |  |
| OXA-2-F | GCCAAAGGCACGATAGTTGT | *bla*OXA-2 | 700 |  |  |
| OXA-2-R | GCGTCCGAGTTGACTGCCGG |  |  |  |  |
| OXA-10-F | TCTTTCGAGTACGGCATTAGC | *bla*OXA-10 | 760 |  |  |
| OXA-10-R | CCAATGATGCCCTCACTTTCC |  |  |  |  |
| SHV-S-F | ATGCGTTATATTCGCCTGTG | *bla*SHV | 862 | [39] |  |
| SHV-S-R | AGCGTTGCCAGTGCTCGATC |  |  |  |  |
| TEM-S-F | ATGAGTATTCAACATTTCCG | *bla*TEM | 858 |  |  |
| TEM-S-R | CCAATGCTTAATCAGTGAGG |  |  |  |  |
| ERIC2 | AAGTAAGTGACTGGGGTGAGCG |  |  | [40] |  |
| adk-P1 | TCATCATCTGCACTTTCCGC | adk | 583 | [41] |  |
| adk-P2 | CCAGATCAGCGCGAACTTCA |  |  |  |  |
| fumC-P1 | TCACAGGTCGCCAGCGCTTC | fumC | 806 |  |  |
| fumC-P2 | TCCCGGCAGATAAGCTGTGG |  |  |  |  |
| gyrB-P1 | TCGGCGACACGGATGACGGC | gyrB | 911 |  |  |
| gyrB-P2 | GTCCATGTAGGCGTTCAGGG |  |  |  |  |
| icd-P1 | ATGGAAAGTAAAGTAGTTGTTCCGGCACA | icd | 878 |  |  |
| icd-P2 | GGACGCAGCAGGATCTGTT |  |  |  |  |
| mdh-P1 | AGCGCGTTCTGTTCAAATGC | mdh | 932 |  |  |
| mdh-P2 | CAGGTTCAGAACTCTCTCTGT |  |  |  |  |
| purA-P1 | TCGGTAACGGTGTTGTGCTG | purA | 816 |  |  |
| purA-P2 | CATACGGTAAGCCACGCAGA |  |  |  |  |
| recA-P1 | ACCTTTGTAGCTGTACCACG | recA | 780 |  |  |
| recA-P2 | AGCGTGAAGGTAAAACCTGTG |  |  |  |  |
|  |  |  |  |  |  |
